# Parallel evolution of *vgsc* mutations at domains IS6, IIS6 and IIIS6 in pyrethroid resistant *Aedes aegypti* from Mexico

**DOI:** 10.1038/s41598-018-25222-0

**Published:** 2018-04-30

**Authors:** Karla Saavedra-Rodriguez, Farah Vera Maloof, Corey L. Campbell, Julian Garcia-Rejon, Audrey Lenhart, Patricia Penilla, Americo Rodriguez, Arturo Acero Sandoval, Adriana E. Flores, Gustavo Ponce, Saul Lozano, William C. Black

**Affiliations:** 1 Arthropod-borne and Infectious Disease Laboratory, Department of Microbiology, Immunology and Pathology, Colorado State University, Fort Collins, Colorado, United States of America; 2 Laboratorio de Arbovirología, Centro de Investigaciones Regionales Dr. Hideyo Noguchi, Universidad Autónoma de Yucatán, Mérida, Mexico; 3 Division of Parasitic Diseases and Malaria, Centers for Disease Control and Prevention, Atlanta, Georgia United States of America; 4 Centro Regional de Investigacion en Salud Publica, Instituto Nacional de Salud Publica, Tapachula, Chiapas Mexico; 5 Laboratorio de Entomologia Medica, Facultad de Ciencias Biologicas, Universidad Autonoma de Nuevo Leon, Monterrey, Mexico

## Abstract

*Aedes aegypti* is the primary urban mosquito vector of viruses causing dengue, Zika and chikungunya fevers –for which vaccines and effective pharmaceuticals are still lacking. Current strategies to suppress arbovirus outbreaks include removal of larval-breeding sites and insecticide treatment of larval and adult populations. Insecticidal control of *Ae. aegypti* is challenging, due to a recent rapid global increase in knockdown-resistance (kdr) to pyrethroid insecticides. Widespread, heavy use of pyrethroid space-sprays has created an immense selection pressure for kdr, which is primarily under the control of the voltage-gated sodium channel gene (*vgsc*). To date, eleven replacements in *vgsc* have been discovered, published and shown to be associated with pyrethroid resistance to varying degrees. In Mexico, F1,534C and V1,016I have co-evolved in the last 16 years across *Ae. aegypti* populations. Recently, a novel replacement V410L was identified in Brazil and its effect on *vgsc* was confirmed by electrophysiology. Herein, we screened V410L in 25 *Ae. aegypti* historical collections from Mexico, the first heterozygote appeared in 2002 and frequencies have increased in the last 16 years alongside V1,016I and F1,534C. Knowledge of the specific *vgsc* replacements and their interaction to confer resistance is essential to predict and to develop strategies for resistance management.

## Introduction

Pyrethroids are the most common class of insecticides used to suppress adult populations of *Aedes aegypti*, the principal vector of dengue, chikungunya, Zika and yellow fever viruses. The lack of vaccines for most of these arboviruses results in a tremendous reliance on chemical control. Unfortunately, intense application of pyrethroids has resulted in pyrethroid resistance in *Ae. aegypti* populations worldwide^1–5^. A major mechanism underlying pyrethroid resistance is known as knockdown resistance (kdr), which is caused by mutations in the voltage-gated sodium channel (*vgsc*)^6^. Knowledge of the specific *vgsc* mutations that confer resistance is essential to predict the rise of pyrethroid resistance and to develop strategies for resistance management.

Globally, eleven *vgsc* mutations have been identified in *Ae. aegypti* and in most cases, have been linked to conferring some degree of pyrethroid resistance. These include G923V, L982W, I1,011 M and V1,016 G first identified in 2003^7^, I1,011 V and V1,016I in 2007^8^, D1,763Y in 2009^9^, S989P and F1,534C in 2010^10,11^, T1,520I in 2015^12^ and V410L in 2017^13^. These kdr mutations are usually confined to specific geographic areas and co-occurrence of certain mutations is a common phenomenon sometimes associated with higher levels of phenotypic resistance^14^.

To date, only five mutations have been functionally confirmed to reduce *vgsc* sensitivity to pyrethroids, including S989P (IIL5–6), I1,011 M, V1,016 G (IIS6), F1,534C (IIIS6)^14^ and most recently V410L (IS6)^13^. Computer modeling predicts that pyrethroids bind to two homologous lipid exposed interfaces between domains: one binding site (PYR-1) is formed by the linker helix connecting S4 and S5 in domain II (IIL45) and domains IIS5, IIS6, and IIIS6, and the second binding site (PYR-2) is formed by the linker helix connecting S4 and S5 in domain I (IL45) and domains IS5, IS6, and IIS6^14–17^.

In Mexico, pyrethroid-resistant *Ae. aegypti* populations carry at least two *vgsc* mutations, V1,016I which is linked to permethrin survival^8^ and F1,534C. Interestingly, F1,534C reduces permethrin binding to *vgsc* channels whereas V1,016I has no effect in pyrethroid binding^14^. However, both replacements have co-evolved in Mexican populations; V1,016I and F1,534C allele frequencies have increased in the last 16 years^18^ and F1,534C has reached fixation in several locations in Southern Mexico^19^. The co-occurrence of V1,016I and F1,534C has been reported in several countries in Latin America, including Brazil^20,21^, Venezuela^22^, Colombia^23^, Martinique Island^24^, Puerto Rico^25^, Grand Cayman Islands^4^, Cuba^26^, Haiti^27^ and Jamaica^28^.

**Figure 1:**
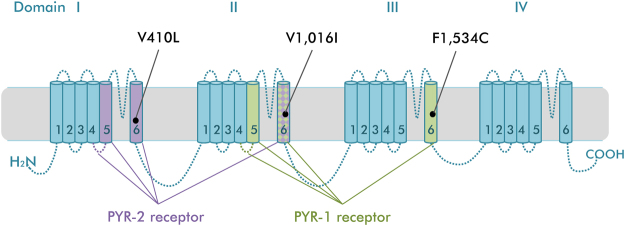
Physical location of V410L, V1,016I and F1,534C replacements in the *vgsc*. The domain segments and interlink helices forming the pyrethroid receptor sites 1 (green) and 2 (purple) are shown.

Recently, a novel mutation V410L in domain IS6 was identified in a pyrethroid resistant laboratory strain of *Ae. aegypti* from Brazil^13^. Alone or in combination with F1,534C, V410L reduced the sensitivity of mosquito sodium channels expressed in *Xenopus* oocytes to both type I (eg. permethrin) and II pyrethroids (eg. deltamethrin). Interestingly, authors did not detect this mutation in field populations from Pernambuco, Brazil, concluding the novel mutation may not yet be widespread in nature. In contrast, we identified high frequencies of V410L alongside V1,016I and F1,534C in a genome-wide association study of *Ae. aegypti* from Mexico. To extend this observation, we screened the frequencies of V410L in different temporal and geographical *Ae. aegypti* collections made over the last 16 years in Mexico. We compared the evolution and linkage disequilibrium of V410L alongside the V1,016I and F1,534C replacements, which occur in different domains IS6, IIS6 and IIIS6, respectively (Fig. 1). In addition, we determined the phenotype/genotype association in a field population exposed to permethrin and deltamethrin.

## Results

### V410L in *Ae. aegypti* collections from Mexico

**Figure 2:**
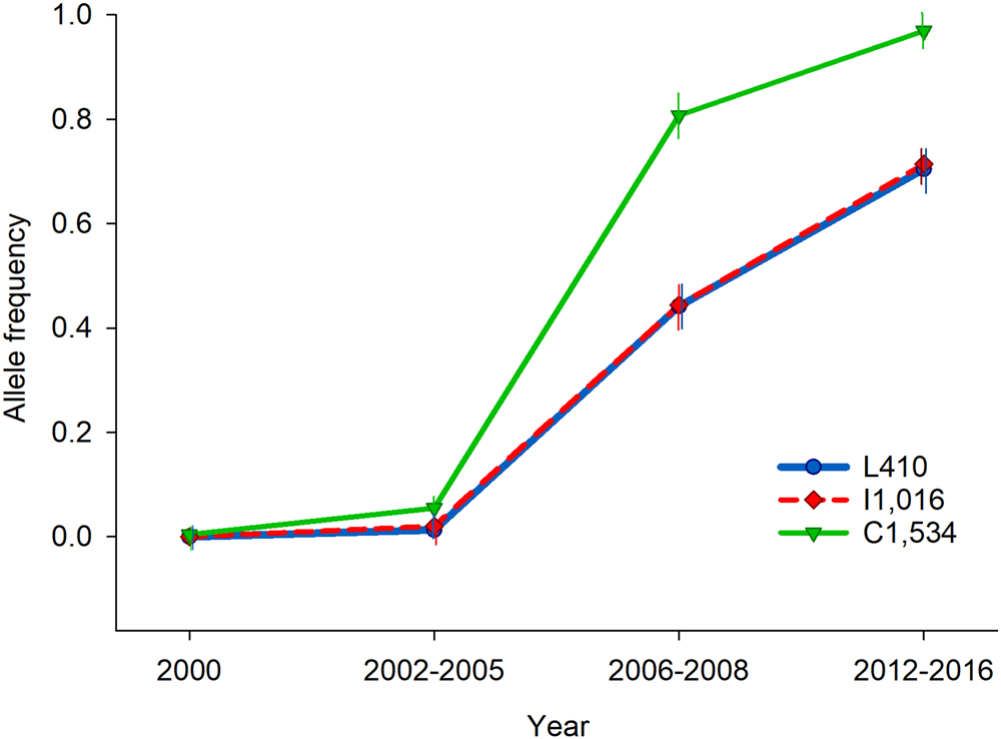
L410, I1,016 and C1,534 allele frequencies in 25 *Ae. aegypti* collections from Southern Mexico. Allele frequencies are plotted in four periods of time: 2000 (n = 233), 2002–2005 (n = 346), 2006–2008 (n = 223) and 2012–2016 (n = 374).

We determined the V410L genotypes for 1,176 mosquitoes collected from six field locations in Mexico from 2000–2016. The V410L genotype counts and calculated allele frequencies are shown in Table 1. V410L first appeared in a heterozygous individual mosquito in 2002 in Coatzacoalcos. By 2008, L410 allele frequencies ranged from 0.27–0.65 across collections. By 2012, L410 frequencies ranged from 0.56 to 0.84 in collections from the State of Veracruz. By 2014, Tapachula and Merida had allele frequencies of 0.57 and 0.9, respectively. V410L genotype frequencies were in Hardy-Weinberg equilibrium in 13 out of 14 collections where V410L was segregating. The exception was in Coatzacoalcos 2008 that had a significant deficiency of LL_410_ homozygotes (F_IS_ = −0.382). Figure 2 shows the allele frequencies of L410, I1,016 and C1,534 at four-time points: 2000 (n = 233), 2002–2005 (n = 346), 2006–2008 (n = 223) and 2012–2016 (n = 374) for all locations. As previously observed for I1,016 and C1,534^19^, L410 also increased in frequency from 2000 to 2016, noting that at each of these time points L410 and I1,016 alleles changed frequencies in parallel from 0.00 to 0.71 (Fig. 2).

**Table 1:** V410L genotypes and allele frequencies in 25 collections from Mexico.

Site	Year	n	V410L genotype			L410 allele frequency and 95% HDI			F_IS_
			VV	VL	LL	Freq.	lower	upper	
Poza Rica	2000	46	46	0	0	0.00	0.0003	0.04	
	2003	47	47	0	0	0.00	0.0003	0.04	
	2008	39	5	17	17	0.65	0.5400	0.75	0.04
	2012	37	3	12	22	0.76	0.6476	0.84	0.12
Martinez de la Torre	2000	46	46	0	0	0.00	0.0003	0.04	
	2002	42	42	0	0	0.00	0.0003	0.04	
	2003	30	24	5	1	0.12	0.0586	0.22	0.19
	2008	48	9	25	14	0.55	0.4521	0.65	−0.05
	2012	44	0	14	30	0.84	0.7555	0.90	−0.18
Zempoala	2000	47	47	0	0	0.00	0.0003	0.04	
	2002	47	47	0	0	0.00	0.0003	0.04	
	2003	30	30	0	0	0.00	0.0004	0.06	
	2012	52	5	18	29	0.73	0.6246	0.79	0.12
Coatzacoalcos	2002	50	48	2	0	0.02	0.0062	0.07	−0.02
	2003	48	48	0	0	0.00	0.0003	0.04	
	2008	48	21	26	0	0.27	0.1964	0.37	−0.38
	2012	45	9	22	14	0.56	0.4626	0.66	0.01
Tapachula	2000	48	48	0	0	0.00	0.0003	0.04	
	2006	42	28	11	3	0.20	0.1307	0.30	0.19
	2014	47	7	26	14	0.57	0.4732	0.67	−0.13
	2016	96	15	40	41	0.64	0.5653	0.70	0.10
Merida	2000	47	47	0	0	0.00	0.0003	0.04	
	2005	48	48	0	0	0.00	0.0003	0.04	
	2007	47	7	30	10	0.53	0.4315	0.63	−0.28
	2013	50	0	10	40	0.90	0.8256	0.94	−0.11
New Orleans		48	48	0	0	0.00			

The site, year, sample size, genotype frequency, L410 allele frequency, 95% high density intervals (HDI) and inbreeding coefficients (F_IS_). L = resistant allele, V = susceptible allele.

Pairwise linkage disequilibrium analyses were performed between locus 410-1,016, 410–1,534 and 1,016–1,534. Table 2 shows the linkage disequilibrium coefficients (R_ij_), χ^2^ and associated probabilities obtained between pairwise loci. Fourteen out of 25 collections had alleles segregating at loci 410–1,016 with R_ij_ values ranging between 0.53–0.99 among collections; overall R_ij_ was 0.96 (p = 0.0001). For loci 410–1,534, only five collections had mutant alleles segregating, and four were in significant linkage disequilibrium with R_ij_ ranging from 0.33 to 0.99; the overall R_ij_ coefficient was 0.76 (p = 0.0001). At loci 1,016–1,534, segregating alleles from six collections were in significant linkage disequilibrium, with R_ij_ coefficients ranging from 0.31 to 0.99; overall R_ij_ coefficient was 0.76 (p = 0.0001).

**Table 2:** Linkage disequilibrium coefficients (R_ij_), χ^2^ and associated probabilities between loci 410-1,016, 410-1,534 and 1,016-1,534.

Site	Year	410-1,016			410-1,534			1,016-1,534		
		R_ij_	χ^2^	P	R_ij_	χ^2^	p	R_ij_	χ^2^	p
Poza Rica	2008	0.67	17.3	0.0001	0.41	6.5	0.0106	0.72	20.0	0.0001
	2012	0.61	13.9	0.0002	0.31	3.5	0.0622	—	—	—
Martinez de la Torre	2003	0.53	8.5	0.0035	—	—	—	0.69	14.3	0.0002
	2008	0.99	48.0	0.0001	—	—	—	—	—	—
	2012	0.99	45.0	0.0001	—	—	—	—	—	—
Zempoala	2012	0.81	34.3	0.0001	—	—	—	—	—	—
Coatzacoalcos	2002	0.99	50.0	0.0001	0.99	50.0	0.0001	0.99	50.0	0.0001
	2008	0.99	47.0	0.0001	—	—	—	—	—	—
	2012	0.99	47.0	0.0001	—	—	—	—	—	—
Tapachula	2006	0.99	42.0	0.0001	0.33	4.7	0.0303	0.33	4.7	0.0303
	2014	0.89	37.3	0.0001	—	—	—	—	—	—
	2016	0.99	96.0	0.0001	—	—	—	—	—	—
Merida	2007	0.88	36.0	0.0001	0.96	43.4	0.0001	0.82	31.4	0.0001
	2013	0.82	33.9	0.0001	—	—	—	0.31	4.8	0.0278
Overall population		0.96	1081.7	0.0001	0.76	675.9	0.0001	0.76	676.9	0.0001

### Temporal analysis of tri-locus genotypes

**Figure 3:**
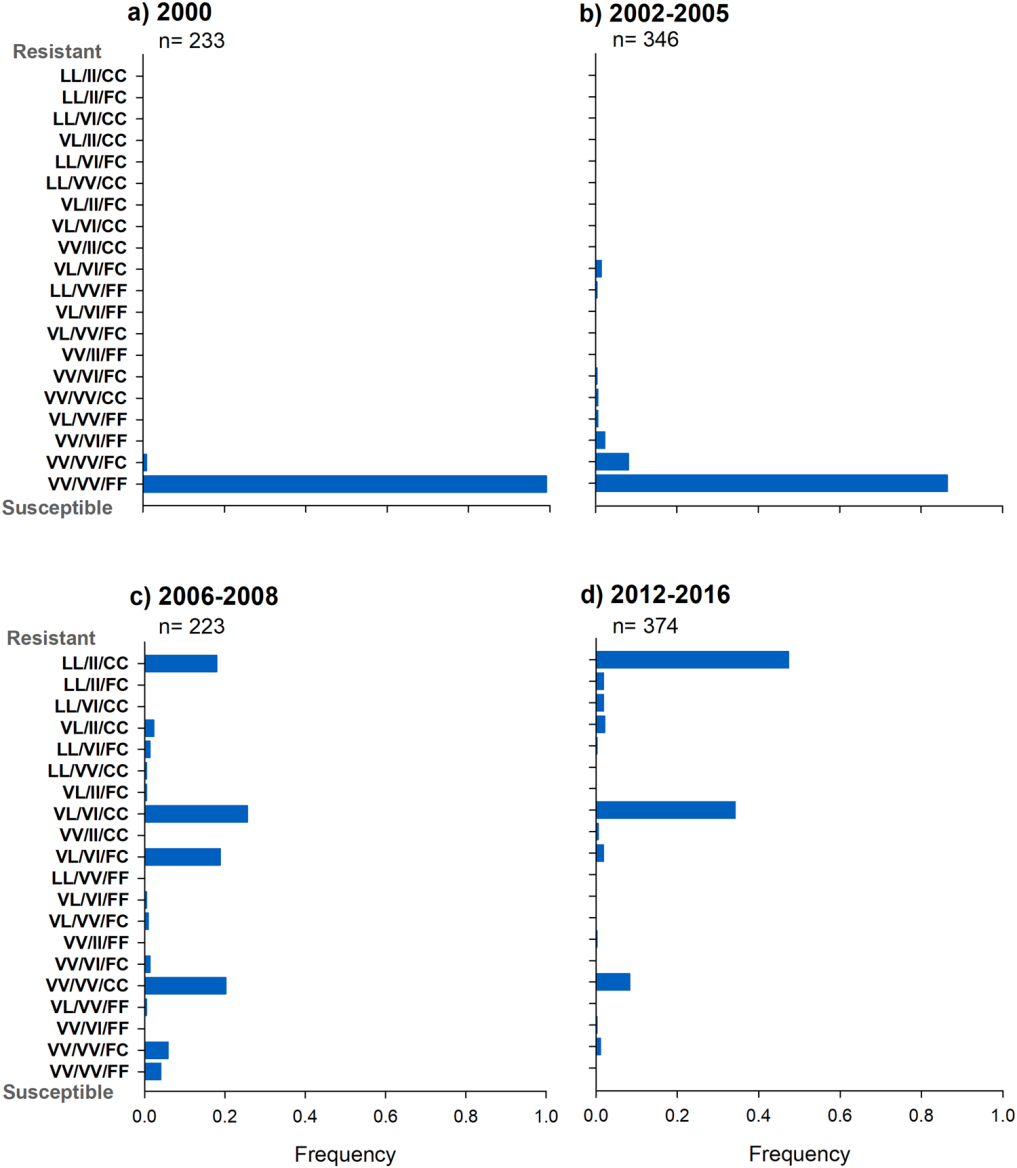
Frequencies of the 20 tri-locus genotypes plotted by periods of time. (**a**) Frequencies in 2000, (**b**) Frequencies in 2002–2005, (**c**) Frequencies in 2006–2008 and (**d**) Frequencies in 2012–2016. The order of the genotypes is 410/1,016/1,534. Resistant allele at 410 = L, 1,016 = I and 1,534 = C. The triple susceptible genotype is at the bottom of each graph whereas the triple resistant genotype is show at the top of each graph.

Out of 27 genotype combinations (3 genotypes at 3 loci), we found 20 tri-locus genotype combinations in 1,176 individual mosquitoes collected from 2000 to 2016. Figure 3 shows the frequency of each of the 20 tri-locus genotype combinations at four-time points: 2000, 2002–2005, 2006–2008 and 2012–2016. In 2000, the triple homozygote susceptible genotype (VV_410_/VV_1,016_/FF_1,534_) occurred at a high frequency (0.99) whereas a genotype including a heterozygote at loci 1,534 (VV_410_/VV_1,016_/FC_1,534_) had a frequency lower than 0.01 (Fig. 3a). By 2002–2005, these genotypes were still the most common (frequencies of 0.86 and 0.08, respectively), however, six additional genotypes including homozygotes at locus 1,534 and heterozygotes at loci 1,016 and 410 occurred at very low frequencies (<0.02) (Fig. 3b). By 2006–2008, the triple homozygote susceptible genotype (VV_410_/VV_1,016_/FF_1,534_) and the 1,534 heterozygotes decreased to frequencies lower than 0.06. Twelve additional combinations occurred, the four most frequent genotypes were VV_410_/VV_1,016_/CC_1,534_ (frequency = 0.2), VL_410_/VI_1,016_/FC_1,534_ (0.18), VL_410_/VI_1,016_/CC_1,534_ (frequency = 0.26) and the triple resistant homozygote LL_410_/II_1,016_/CC_1,534_ (frequency = 0.18) (Fig. 3c). Observed frequencies of these genotypes were significantly higher than expected (Supplementary Table S1). By 2012–2016, the triple homozygote susceptible genotype was no longer detected, and the most common genotype combinations were the triple resistant homozygote (frequency = 0.47) and VL_410_/VI_1,016_/CC_1,534_ (frequency = 0.34) (Fig. 3d). Observed frequencies of these genotypes were in significant excess. In the same period, very low frequencies of resistant homozygotes at locus 410 occurred independently of 1,016 (LL_410_/VV_1,016_/CC_1,534_ or VV_410_/II_1,016_/CC_1,534_ and LL_410_/VV_1,016_/FC_1,534_ or VV_410_/II_1,016_/FC_1,534_). Also, genotypes including heterozygotes at locus 410 and homozygotes at locus1,016 and vice versa (VL_410_/II_1,016_/CC_1,534_ or VL_410_/II_1,016_/FC_1,534_ and LL_410_/VI_1,016_/CC_1,534_ or LL_410_/VI_1,016_/FC_1,534_) were observed at 10-fold lower frequencies (~7 individuals) than expected (Supplementary Table S1).

### Association of V410L with pyrethroid resistance

L410 was present at a frequency of 0.69 in Viva Caucel mosquitoes used for our phenotype/genotype association study. We used a dose of permethrin or deltamethrin to discriminate three phenotypes: knockdown resistant, recovered and dead. The susceptible genotype at locus 410 was VV_410_, heterozygote was VL_410_ and resistant was LL_410_. Table 3 shows the outcomes of mosquitoes carrying a specific genotype in terms of response to pyrethroid treatment. For permethrin, 53% of the resistant homozygotes (LL_410_) were knockdown resistant, 40% recovered and only 7% died. In the heterozygote group (VL_410_) 4% was knockdown resistant, 28% recovered and 64% died following permethrin exposure. For deltamethrin, 72% of the resistant homozygotes (LL_410_) were knockdown resistant whereas 23% recovered and only 5% died. Note that the phenotype outcome is very similar between genotypes at loci 410 and 1,016. The most striking difference at locus 1,534, is that more than 92% of the heterozygotes died after exposure to permethrin or deltamethrin. Across all analyses, strong correlations were detected between phenotype and genotype.

**Table 3:** Phenotype and genotype at loci 410, 1,016 and 1,534 analyzed separately in mosquitoes from Viva Caucel treated with permethrin or deltamethrin.

Loci	Genotype	Permethrin			Deltamethrin		
		Knockdown resistant	Recovered	Dead	Knockdown resistant	Recovered	Dead
		n = 94	n = 95	n = 95	n = 111	n = 67	n = 92
V410L	LL	87 (53%)	66 (40%)	12 (7%)	86 (72%)	28 (23%)	6 (5%)
	VL	4 (5%)	28 (32%)	56 (64%)	24 (20%)	35 (29%)	52 (47%)
	VV	2 (7%)	1 (3%)	27 (90%)	1 (1%)	4 (3%)	34 (87%)
	*p*	4.0 × 10^−30^			6.1 × 10^−26^		
V1,016I	II	90 (54%)	66 (40%)	11 (7%)	86 (70%)	27 (22%)	9 (7%)
	VI	3(3%)	28 (32%)	57 (65%)	24 (20%)	36 (30%)	52 (46%)
	VV	1 (3%)	1 (3%)	27 (93%)	1 (1%)	4 (3%)	31 (86%)
	*p*	1.4 × 10^−32^			1.2 × 10^−23^		
F1,534C	CC	90 (37%)	95 (39%)	58 (24%)	111 (56%)	65 (33%)	24 (12%)
	FC	3 (8%)	0 (0%)	36 (92%)	0 (0%)	2 (1%)	61 (97%)
	FF	1 (50%)	0 (0%)	1 (50%)	0 (0%)	0 (0%)	6 (100%)
	*p*	7.9 × 10^−15^			6.8 × 10^−35^		

The percentage of knockdown resistant, recovered and dead mosquitoes within the genotype group is shown in parenthesis. The *p* value corresponds to a 3 × 3 table contingency analysis performed for each locus.

### Association of tri-locus genotypes with pyrethroid resistance

**Figure 4:**
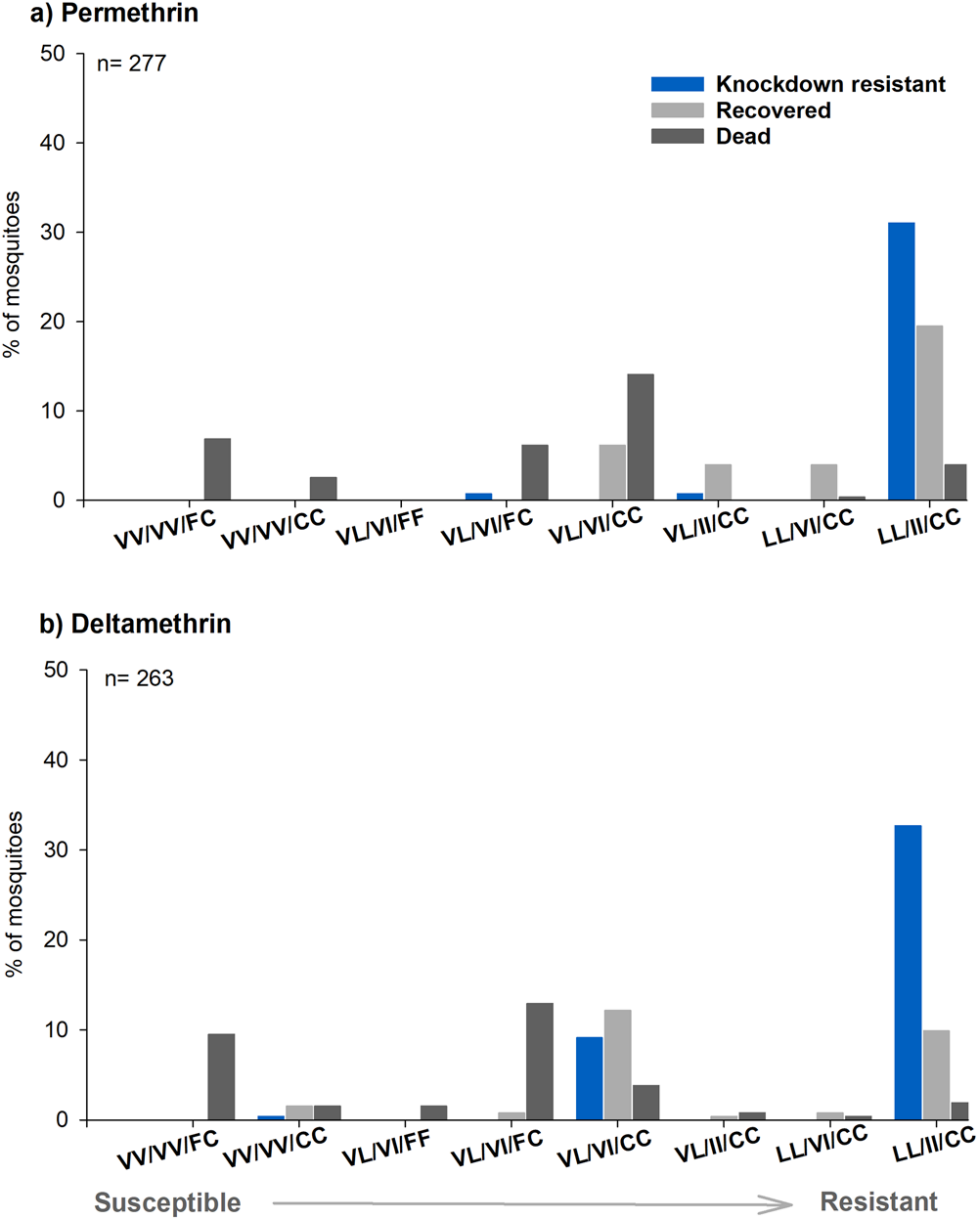
Frequencies of tri-locus genotypes in knockdown resistant, recovered and dead mosquitoes following (**a**) permethrin or (**b**) deltamethrin exposure. The order of the genotypes is shown for locus 410/1,016/1,534 respectively. Resistant allele at loci 410 = L, 1,016 = I and 1,534 = C. The triple susceptible genotype is closer to the y axis whereas the triple resistant genotype is shown on the far right side of the x axis.

Because our results indicated that L410, I1,016 and C1,534 do not occur independently, we analyzed the phenotype outcome by tri-locus genotype combinations. In the Viva Caucel population, 13 tri-locus genotype combinations were identified. Figure 4 shows the distribution of knockdown resistant, recovered and dead mosquitoes for the eight most common tri-locus genotype combinations. The presence of resistant alleles in the tri-locus homozygote genotype is strongly associated with knockdown resistance and recovery for both permethrin and deltamethrin (Fig. 4a,b). Wild type homozygotes at locus 410 and 1,016 in presence of FC_1,534_ or CC_1,534_ were associated with the dead phenotype. The double heterozygotes at locus 410 and 1,016 in presence of FF_1,534_ or FC_1,534_ were also associated with the dead phenotype for both pyrethroids. The double heterozygote at locus 410 and 1,016 with CC_1,534_ (VL_410_/VI_1,016_/CC_1,534_) was associated with the dead phenotype for permethrin exposure but was associated with knockdown resistance and recovery in the deltamethrin exposure group.

## Discussion

Different replacements at residue V410 have been reported in the *vgsc* of several pyrethroid resistant insect species. Specifically, V410L was associated with deltamethrin resistance in the common bed bug *Cimex lectularis*^29^. However, replacements V410M in the tobacco budworm^30^ and V410A, V410G and V410M in the earworm^31^ have also been reported. In *Ae. aegypti*, V410L was recently detected in a pyrethroid resistant insectary strain from Brazil^13^, which demonstrated that V410L alone or in combination with the F1,534C reduced the sensitivity of mosquito sodium channels expressed in *Xenopus* oocytes to both type I and II pyrethroids. In the same study, V410L was not detected in a small field survey in the State of Pernambuco, Brazil, and authors suggested that V410L was not yet widespread in the field. Importantly, our results show that V410L has existed for at least 16 years in Mexico, the first heterozygote was detected in 2002 in Coatzacoalcos, Veracruz, and has increased gradually to high frequencies in 2016 (up to 0.92). Interestingly, we found L410 to be in greater linkage disequilibrium with I1,016 than with C1,534. Our previous study measured linkage disequilibrium between I1,016 and C1,534 and we proposed a sequential model, wherein C1,534 first occurred (providing low levels of resistance) and then the replacement I1,016 occurred with this haplotype, providing even higher levels of resistance. V410L challenges this sequential model, in which both V410L and V1,016I might have occurred independently on a C1,534 haplotype and then became *cis* to C1,534 by recombination. An alternative model assumes the three mutations arose independently at very low frequencies, and then by two recombination events, came to occur in a *cis* arrangement. Our results indicate that C1,534 was at a frequency of 0.004 in collections from 2000 whereas L410 and I1,016 were below limits of detection. By 2002–2005, C1,534 was at higher frequency (0.055), while L410 and I1,016 appeared at lower frequencies (0.013 and 0.02, respectively). During this period, we identified heterozygotes at 410 and 1,016 occurring independently in a FF_1,534_ background. Also, the triple heterozygote was identified in low frequencies (5/346). By 2006–2008 heterozygotes VL_410_ and VI_1,016_ on a FC_1,534_ heterozygote or CC_1,534_ homozygote were favored by selection, whilst mutant genotypes at 410 and 1,016 never occurred independently or were at very low frequencies.

A recent study found that V410L and F1,534C occurred without V1,016I in a deltamethrin resistant laboratory strain originated from Rio de Janeiro^13^. In contrast, we show large linkage disequilibrium between L410 and I1,016, except among very few individuals collected early in 2002–2005; however, this genotype combination was no longer detected in following years. Whether L410 remains independent of I1,016 in Brazil will provide evidence of the mutations arising independently at a local level in the sequential model. However, I1,016 and C1,534 are already widespread in several regions of Brazil, and due to high migration rates among *Ae. aegypti* populations, we would expect I1,016 and C1,534 to recombine with L410-C1,534 in future years. However, an alternative scenario is that as in Mexico, L410 is already present at high frequencies in Brazilian collections previously genotyped with I1,016-C1,534 but simply has not yet been detected.

The selection of the triple homozygote resistant genotypes detected in our data suggests higher fitness of this genotype in the presence of pyrethroids. The role of L410 and C1,534 in conferring pyrethroid resistance was determined in Haddi *et al*. 2016^13^. L410 alone or in combination with C1,534 confers high levels of resistance, however, it remains to be seen if it is fit in the field. We found 4 out of 1,176 individuals with L410 and C1,534 occurring independently (in the absence of I1,016), and this genotype became extinct in Mexican populations. In our phenotype and genotype studies, the triple homozygote resistant individuals had better survival (knockdown resistance and recovery) following either permethrin or deltamethrin exposure. One particular genotype, VL_410_/VI_1016_/CC_1,534_ had different outcome depending of the specific pyrethroid, with this genotype associated with dead in mosquitoes following permethrin exposure. In contrast, this genotype was mostly associated with survival (knockdown and recovery) in mosquitoes exposed to deltamethrin. Apparently, the presence of heterozygotes at loci 410 and 1,016 was sufficient for deltamethrin survival.

F1,534C is located in the PYR-1 receptor site and is responsible for reducing vgsc sensitivity to permethrin. Although residue 1,016 is located in the PYR-1 receptor site, only the V1,016G replacement occurring in *Ae. aegypti* from Asia reduces vgsc whilst the V1,016I replacement found in the Americas does not^16^. In contrast, V410L is located in DIS6 but does not form part of the PYR-2 receptor site. It has been suggested that the reduction of sodium channel sensitivity to permethrin and deltamethrin by V410L might result from changes in the gating properties of vgsc without inhibiting molecule docking^13^. Because pyrethroids prefer to bind to sodium channels in the open state, kdr mutations that deter the open state would counteract the pyrethroid effects^13^. In recent structural models, pyrethroids make multiple contacts with helices IIL45, IIS5, IIS6, and IIIS6, as well as IL45, IS5, IS6, and IIS6 that would maintain vgsc in an open state^17,32^. Simultaneous binding of pyrethroids to both PYR-1 and PYR-2 is thought to effectively prolong the opening of vgsc^14^. It is possible that co-occurrence of V410L and V1,016I, although in different receptor sites, provide fitness advantages in the presence of pyrethroids, thus favoring co-selection. The interaction of both mutations in electrophysiology experiments will address if the co-occurrence of these mutations is compensatory or synergistic in the presence of pyrethroids.

## Methods

### Detection of V410L and genotyping

Primer positions at exon 9 and 10 of *vgsc* in *Ae. aegypti* are shown in Supplementary Figure 1. We used primers 410fw 5′-GATAATCCAAATTACGGGTATAC-3′ and 410rev 5′-TTCTTCCTCGGCGGCCTCTT-3′ to amplify a ~500 bp region that flanked exon 9, intron 9–10 (~239 bp) and exon 10. PCR reactions were performed using 12.5 µl of GoGreenTaq (Promega, Madison WI), 11.65 µl H_2_0, 1.1 µl of each primer (at 50 pmol/µl) and 1 µl genomic DNA (~25 ng). PCR conditions were 3 min at 94 °C, followed by 35 cycles of 30 sec at 94 °C, 30 sec at 60 °C and 1 min at 72 °C and a final extension of 5 min at 72 °C. Products were purified using the MinElute PCR purification columns (Qiagen, Hilden Germany) and sequenced using the primers 410_ex10fw 5′-TACGATCAGCTGGACCGTGG and 410rev targeting a fragment of 174 bp in exon 10. Sequences were analyzed using the Geneious software (Biomatters Inc, Newark NJ).

Following identification of the G (GTA = Val) and T (TTA = Leu) alleles at locus 410, we designed an allele-specific PCR system to detect individual genotypes by melting curve analysis^8^. Each reaction contained 50 µM of each forward primer V410fw (5′-GCGGGCAGGGCGGCGGGGGCGGGGCCATCTTCTTGGGTTCGTTCTACCGTG-3′), and L410fw (5′-GCGGGCATCTTCTTGGGTTCGTTCTACCATT-3′) and 100 µM of reverse primer 410rev (5′-TTCTTCCTCGGCGGCCTCTT-3′), 10 µl Sybr Green Master (BioRad, Hercules CA), 9.7 µl ddH_2_0 and 1 µl of genomic DNA (~25 ng). PCR and melting curve analysis was run in a CFX-96 (BioRad) following 3 min at 95 °C, 39 cycles of 10 sec at 95 °C, 10 sec at 60 °C, 30 sec at 72 °C followed by a melting curve from 65 °C to 95 °C with increments of 0.2 °C during 10 sec. The products consisted of  a single 133 bp amplicon for a VV_410_ homozygote, a single 113 bp amplicon for a LL_410_ homozygote (resistant) and the presence of both amplicons in heterozygote individuals (VL_410_) (Supplementary Figure 2).

### Mosquito collections

We determined the V410L genotypes for 1,176 mosquitoes collected from six field locations in Mexico from 2000–2016 (Supplementary Table S2 and Supplementary Figure 3). Four of these sites were in the State of Veracruz (Eastern Mexico). Tapachula is in the State of Chiapas in Southwestern Mexico and borders Guatemala, while Merida is in the Yucatan peninsula in Southeastern Mexico. The V410L database was completed with genotypes at loci 1,016 and 1,534^19,25^.

### Allele frequencies and linkage disequilibrium analysis

V410L allele frequencies were calculated from genotypic frequencies following Garcia *et al*.^18^. The 95% high density intervals (HDI 95%) around these frequencies were calculated in WINBugs2.0 following 1,000,000 iterations. Departure from Hardy-Weinberg expectations were expressed as Wright’s inbreeding coefficient (F_IS_) and a χ^2^ test was used to test the hypothesis F_IS_ = 0 (d.f. = 1). Pairwise linkage disequilibrium between alleles at loci 410 and 1,016 or between loci 410 and 1,534 was calculated using LINKDIS following Vera-Maloof *et al*.^19^. Composite disequilibrium between resistant alleles was tested and a χ^2^ test determined if significant disequilibrium existed among alleles at both loci.

### V410L association with pyrethroid resistance

To determine phenotype/genotype associations, we used the Viva Caucel (long −89.71827, lat 20.99827) collection from Yucatan, Mexico made in 2011. First, we calculated the permethrin and deltamethrin (Chem Service) lethal concentration that killed 50% of the mosquitoes (LC_50_) in 3–4 days old adults of the F_3_ generation. The insecticide treatment consisted of a 1 h exposure in an insecticide coated bottle, transfer of mosquitoes to recovery chambers and mortality scored at 24 h after treatment. We assessed the levels of permethrin and deltamethrin resistance in Viva Caucel relative to the New Orleans (NO) reference strain. The permethrin LC_50_ was 47.9-fold higher than NO (26.5 µg vs 0.55 µg, respectively) and the deltamethrin LC_50_ was 47.6-fold higher than NO (10.49 µg vs 0.22 µg).

Once the LC_50_ was calculated, six to 10 groups of 50 mosquitoes at a time were exposed to 25 µg of permethrin or 3 µg of deltamethrin coated in 250 mL Wheaton bottles during 1 h. Immediately following exposure, active (knockdown resistant) and inactive mosquitoes were transferred to separate containers. To ensure correct categorization, we phenotyped mosquitoes 4 h after treatment. We observed activity and if the mosquitoes were capable of flight, they were scored as ‘knockdown resistant’. In the inactive group we separated the newly recovered mosquitoes from the inactive mosquitoes and scored them as ‘recovered’ and ‘dead’, respectively. Supplementary Table 3 shows the total number of mosquitoes exposed to permethrin and deltamethrin and the distribution between the three phenotypic categories.

A subsample of mosquitoes from each group was individually frozen; DNA was isolated by the salt extraction method^33^ and resuspended in 150 µl of TE buffer (10 mM Tris-HCl, 1 mM EDTA pH 8.0). For the Viva Caucel mosquitoes exposed to permethrin we randomly selected 95 knockdown-resistant, 95 recovered and 95 dead mosquitoes. For deltamethrin we randomly selected 111 knockdown-resistant, 67 recovered and 92 dead mosquitoes. We conducted genotyping at locus 410 using the V410L melting curve system described above. For V1,016I and F1,534C genotypes, we used previously described methodologies^8,11^. A contingency table was used to test for association between the phenotypes (knockdown resistant, recovered and dead) and genotypes (mutant homozygote, wild type homozygote, and heterozygote) at each locus separately (410, 1,016 and 1,534) and for the 27 (3 × 3 × 3) tri-locus genotype combinations.

### Disclaimer

The findings and conclusions in this paper are those of the authors and do not necessarily represent the official position of the Centers for Disease Control and Prevention.

## Electronic supplementary material

Supplementary files
