# Supplementary material for: Parallel evolution of vgsc mutations at domains IS6, IIS6 and IIIS6 in pyrethroid resistant Aedes aegypti from Mexico
Source: Sci Rep. 2018 Apr 30;8:6747. doi: 10.1038/s41598-018-25222-0 (PMC5928250; doi:10.1038/s41598-018-25222-0)

Parallel evolution of *vgsc* mutations at domains IS6, IIS6 and IIIS6 in pyrethroid resistant *Aedes aegypti* from Mexico

Karla Saavedra-Rodriguez[1], Farah Vera-Maloof[1], Corey L. Campbell[1], Julian Garcia-Rejon[2], Audrey Lenhart[3], Patricia Penilla[4], Americo Rodriguez[4], Arturo Acero-Sandoval[4], Adriana Flores[5], Gustavo Ponce[5], Saul Lozano[1] and William C. Black IV[1*]

[1] Arthropod-borne and Infectious Disease Laboratory, Department of Microbiology, Immunology and Pathology, Colorado State University, Fort Collins, Colorado, United States of America

[2]Laboratorio de Arbovirología, Centro de Investigaciones Regionales Dr. Hideyo Noguchi, Universidad Autónoma de Yucatán, Mérida, México

[3]Division of Parasitic Diseases and Malaria, Centers for Disease Control and Prevention, Atlanta, Georgia, United States of America

[4]Centro Regional de Investigacion en Salud Publica, Instituto Nacional de Salud Publica, Tapachula, Chiapas, Mexico

[5]Laboratorio de Entomologia Medica, Facultad de Ciencias Biologicas, Universidad Autonoma de Nuevo Leon, Monterrey, Mexico

*Corresponding author: William C. Black IV (William.Black@colostate.edu)

Supplementary Table S1. Frequency of tri-locus genotypes at loci 410/1,016/1,534 in Mexico at four periods of time. Observed and expected frequencies are shown for each genotype; * indicate the genotypes were not observed. Bold numbers shown the genotypes that exceeded expected.

No. of resistant alleles	Tri-locus genotype	2000		2002-2005		2006-2008		2012-2016	
		obs.	exp.	obs.	exp.	obs.	exp.	obs.	exp.
0	VV/VV/FF	231	231.0	299	289.0	9	0.80	0	0.00
1	VV/VV/FC	2	1.99	28	33.6	13	6.69	4	0.16
1	VV/VI/FF	0	0	8	11.9	0	1.28	1	0.01
1	VL/VV/FF	0	0	2	7.6	1	1.26	0	0.01
2	VV/VV/CC	0	0.00	2	0.98	**45**	**14.0**	**31**	**2.5**
2	VV/VI/FC	0	0	1	1.39	3	10.68	0	0.79
2	VV/II/FF	0	0	0	0.12	0	0.51	1	0.02
2	VL/VV/FC	0	0	0	0.89	2	10.59	0	0.76
2	VL/VI/FF	0	0	0	0.31	1	2.02	0	0.06
2	LL/VV/FF	0	0	1	0.05	0	0.50	0	0.01
3	VL/VI/FC	0	0	5	0.04	**42**	**16.9**	7	3.79
3	VV/VI/CC*	0	0	0	0.04	0	22.36	0	12.53
3	VV/II/FC*	0	0	0	0.01	0	4.26	0	0.99
3	VL/VV/CC*	0	0	0	0.03	0	22.16	0	11.97
3	VL/II/FF*	0	0	0	0.00	0	0.81	0	0.08
3	LL/VV/FC*	0	0	0	0.01	0	4.19	0	0.91
3	LL/VI/FF*	0	0	0	0.00	0	0.80	0	0.07
4	VV/II/CC	0	0	0	0.00	0	8.93	2	15.63
4	VL/VI/CC	0	0	0	0.00	**57**	**35.4**	**128**	**59.8**
4	VL/II/FC	0	0	0	0.00	1	6.75	0	4.73
4	LL/VV/CC	0	0	0	0.00	1	8.76	0	14.28
4	LL/VI/FC	0	0	0	0.00	3	6.69	1	4.52
4	LL/II/FF*	0	0	0	0.00	0	0.32	0	0.09
5	VL/II/CC	0	0	0	0.00	5	14.12	8	74.55
5	LL/VI/CC	0	0	0	0.00	0	14.00	7	71.24
5	LL/II/FC	0	0	0	0.00	0	2.67	7	5.64
6	LL/II/CC	0	0	0	0.00	**40**	**5.6**	**177**	**88.9**
	Total	233	233	346	346	223	223	374	374

Supplementary Table S2. ***Aedes aegypti*** **collections from Mexico.** The region, site, year and number of mosquitoes genotyped in this study. Geographical coordinates are published in [18, 19].

Region	Site (City and State)	Year	n
Eastern Mexico	Poza Rica, Veracruz	2000	46
		2003	47
		2008	39
		2012	37
	Martinez de la Torre,Veracruz	2000	46
		2002	42
		2003	30
		2008	48
		2012	44
	Zempoala, Veraruz	2000	47
		2002	47
		2003	30
		2012	52
	Coatzacoalcos, Veracruz	2002	50
		2003	48
		2008	48
		2012	45
Southeastern Mexico	Merida, Yucatan	2000	48
		2005	42
		2007	47
		2013	96
Southwestern Mexico	Tapachula, Chiapas	2000	47
		2006	48
		2014	47
		2016	50

Supplementary Table S3. Phenotypic response of *Ae. aegypti* exposed to an LC_{50} of permethrin (pyrethroid type 1) or deltamethrin (pyrethroid type 2) in a bottle bioassay. After an insecticide exposure of 1 h, active mosquitoes were separated from inactive mosquitoes and transferred to different containers. Following 4 h, three possible phenotypes were scored:' knockdown resistant' were mosquitoes active at 1 h of exposure and still active at 4 h; 'recovered' mosquitoes were initially in the inactive group but recovered activity within 4 h and 'dead' mosquitoes were inactive at 1 h and continued to be inactive at 4 h post-exposure.

Insecticide	Knockdown resistant n	Recovered n	Dead n	Total n
Permethrin 25 µg	108 (18%)	96 (16%)	411 (67%)	615
Deltamethrin 3 µg	149 (38%)	93 (24%)	148 (38%)	390

Supplementary Figure 1. Genomic and amino acid sequences of *vgsc* exons 9 and 10. The *410fw* and *410rev* primers were used to amplify a 500 bp region flanking exon 9 and 10. *V410L_ex10 fw* and *410rev* pair was used for Sanger sequencing. The V410L substitution is highlighted in red and the codon is bordered. Allele-specific melting curve primers (*V410fw* and *L410fw*) and reverse primers (*410rev*) are shown below the underlined genomic sequence. L-GC corresponds to a 26 mer GC rich-tail and S-GC to a 6 mer GC rich-tail attached to the 5'-primer sequence.

```
Exon 9
 Q  C  E  E  G  Y  I  C  L  Q  G  Y  G  D  N  P  N  Y  G  Y  T  S  F
CAATGCGAAGAAGGATATATTTGTTTACAAGGTTATGGAGATAATCCAAATTACGGGTATACAAGTTTC
                              410fw 5’-GATAATCCAAATTACGGGTATAC-3’

 D  T  F  G  W  A  F  L  S  A  F  R  L  M  T  Q  D  F  W  E  N  L  Y
GATACTTTCGGATGGGCATTCTTATCTGCCTTTCGTCTAATGACCCAAGACTATTGGGAGAATCTTTATC

Q  L  intron 9
AACTGgttcgtatccaatgaccgatgtgtgaatgagactgagtattcatgatccccctcctcaacctga
ttcctccagaactccaccgaaaaaatctaccgaccatcatttattaaatctcgatttgtatctttttct
tctgcccaaacaactgcaatcaactttcaaccactacaattatccccactctcccctacacttcaaacc

                                  Exon 10
                                   V  L  R  S  A  G  P  W  H  M  L  F
aaaaccaaccaactcttcgtggtgtgcaaaacagGTGTTACGATCAGCTGGACCGTGGCACATGCTCTTC
                     V410L_ex10 fw 5’-TACGATCAGCTGGACCGTGG-3’

                                    V410
 F  I  V  I  I  F  L  G  S  F  Y  L V/L N  L  I  L  A  I  V  A  M  S
TTCATTGTGATTATCTTCTTGGGTTCGTTCTACCTTGTAAATTTGATCTTGGCCATTGTCGCCATGTCGT
   5’-[L-GC]ATCTTCTTGGGTTCGTTCTACCTTG-3’  =>V410fw (wild type 133 bp)
   5’-[S-GC]ATCTTCTTGGGTTCGTTCTACCTTT-3’  =>L410fw (mutant type 113 bp)

Y  D  E  L  Q  K  R  A  E  E  E  E  A  A  E  E  E  A  L  R intron 10
ACGACGAACTCCAGAAGAAGGCCGAAGAGGAAGAGGCCGCCGAGGAAGAAGCGCTTCGGgtgagcgaatt
                    410rev 3’-TTCTCCGGCGGCTCCTTCTT-’5
```

Supplementary Figure 2. Melting curve peaks obtained for V410L genotypes. Representative patterns for the LL_{410} resistant homozygote (a single peak at 82.5ºC), VV_{410} susceptible homozygote (a single peak at 87ºC) and a VL_{410} heterozygote (at 82.5ºC and 87ºC) mosquito. Amplicon sizes are shown for each allele.

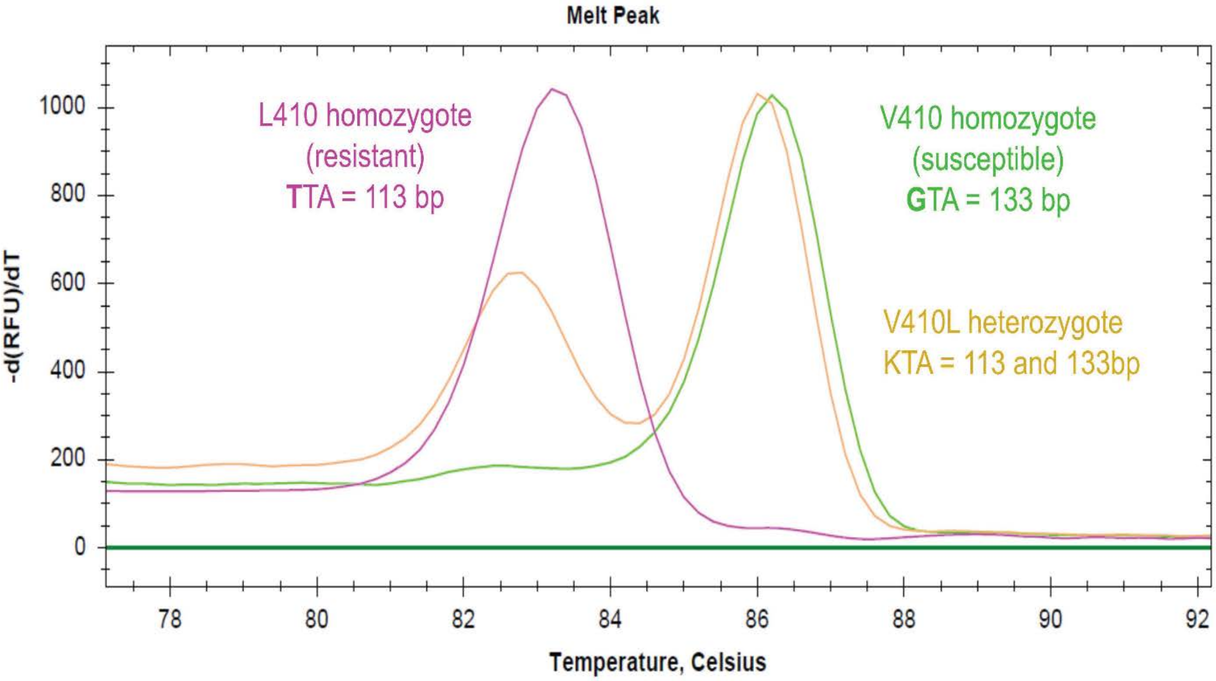

Supplementary Figure 3. Locations of six *Aedes aegypti* collections in the present study.

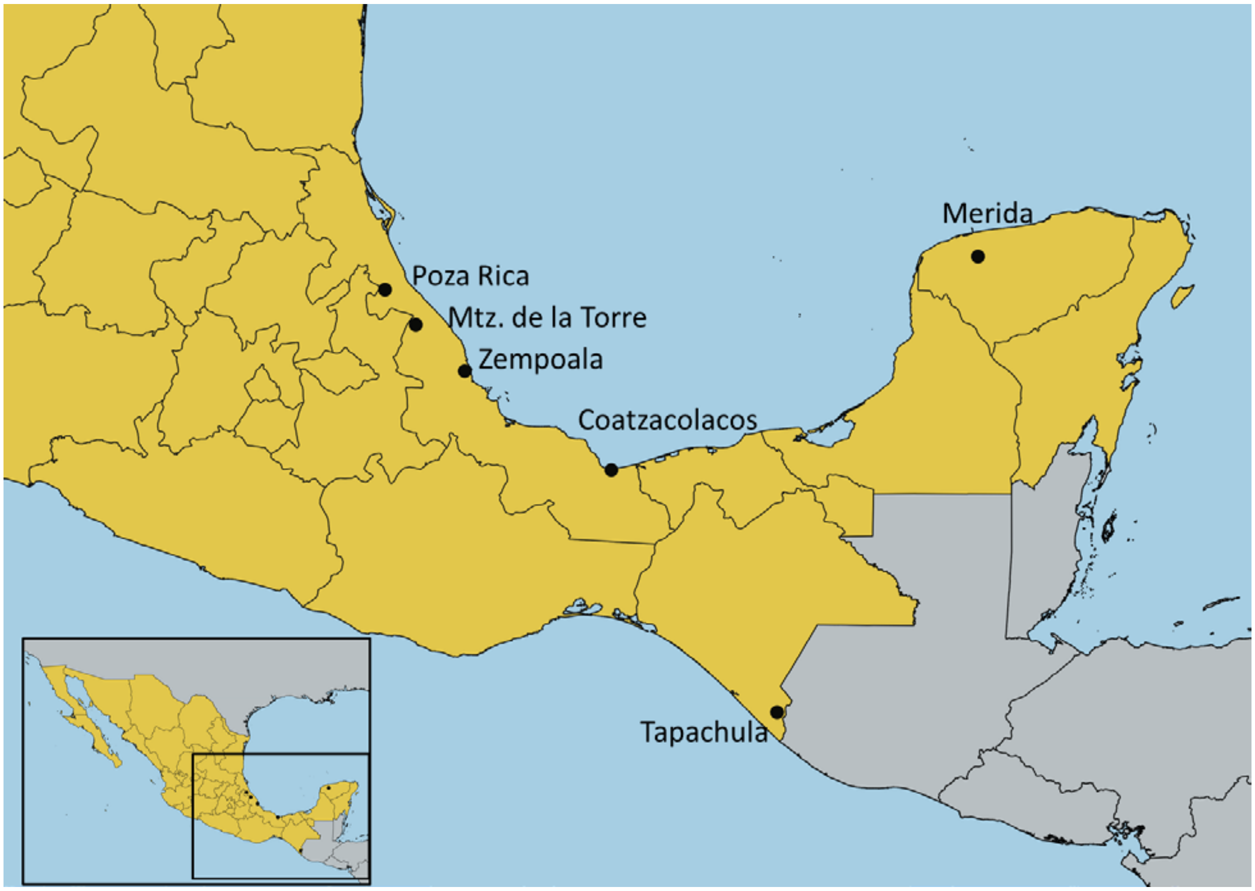

Supplement: Supplementary file 1 — Supplementary files [file 41598_2018_25222_MOESM1_ESM.pdf]
